# 504. Burn Survivor Voices: Three-Year Analysis of a Young Adult Retreat

**DOI:** 10.1093/jbcr/irag033.163

**Published:** 2026-04-06

**Authors:** Margot Merinsky, Daniel W Chacon, Cindy Rutter, Patrick Barton

**Affiliations:** BC Professional Fire Fighters' Burn Fund, Vancouver, BC; Phoenix Society for Burn Survivors, Fresno, CA; 1 Amazing Life, Carlsbad, CA; BC Professional Fire Fighters' Burn Fund, Kelowna, BC

## Abstract

**Introduction:**

Young adult burn survivors often experience a loss of structured psychosocial support after aging out of pediatric burn camps. This transition is marked by isolation, identity uncertainty, and limited access to developmentally appropriate programming. To address this gap, a Canadian-based organization developed a trauma-informed recreational retreat integrating adaptive sports and cross-program participant exchange. This study evaluated psychosocial outcomes across three retreat cycles (2023–2025).

**Methods:**

Post-retreat evaluations were collected from participants (n = 29 unique individuals across three years, with repeat attendees counted once at their first experience). Surveys included Likert-scale ratings of overall satisfaction and open-ended questions about participant experiences, favourite aspects, suggested changes, and the perceived impact of group sessions and peer interactions. Quantitative ratings were summarized descriptively, while qualitative responses underwent thematic analysis by independent reviewers to identify recurring psychosocial outcomes.

**Results:**

Participants consistently reported high satisfaction, with 100% rating the retreat as very good or excellent. Thematic analysis identified three core outcomes:

1. Identity Development & Reconnection — described by 14 of 29 participants and reinforced across multiple responses, e.g., “I realized how much my burn has shaped me, but also how much more I am than my scars.”

2. Peer Support & Validation — the most common theme, noted by 25 of 29 participants and frequently emphasized across evaluations, with many describing the retreat as “family — I wasn’t alone anymore.”

3. Emotional Growth & Readiness — described by 17 of 29 participants, with repeated mentions of increased confidence and clarity, as one participant reflected, “I left feeling stronger and ready to take the next step in my healing.”

These outcomes were consistent across all three retreat cycles (n = 29 unique participants).

**Conclusions:**

A retreat model tailored to young adult burn survivors demonstrates meaningful psychosocial benefits, addressing a critical gap between pediatric and adult support systems. The combination of peer connection, identity exploration, and experiential learning appears to foster resilience and long-term reintegration.

**Applicability of Research to Practice:**

This evaluation highlights a replicable model of young adult aftercare that may inform program design across burn foundations and clinical settings. Integration of developmentally appropriate retreats can extend the continuum of psychosocial support, offering survivors critical opportunities for identity development, peer validation, and emotional growth during early adulthood.

**Funding for the study:**

N/A.

